# The Association of Built Environment and Physical Activity in Older Adults: Using a Citywide Public Housing Scheme to Reduce Residential Self-Selection Bias

**DOI:** 10.3390/ijerph15091973

**Published:** 2018-09-10

**Authors:** Yi Lu, Long Chen, Yiyang Yang, Zhonghua Gou

**Affiliations:** 1 Department of Architecture and Civil Engineering, City University of Hong Kong, Kowloon Tong, Hong Kong, China; yiyayang@cityu.edu.hk; 2 City University of Hong Kong Shenzhen Research Institute, Shenzhen 518057, China; 3 School of Resources and Environmental Sciences, Wuhan University, Wuhan 430072, China; longchen_dona@163.com; 4 Cities Research Institute, School of Environment, Gold Coast Campus, Griffith University, Gold Coast, QLD 4222, Australia; z.gou@griffith.edu.au

**Keywords:** physical activity, built environment, older adults, high-density, walking

## Abstract

Previous studies have documented numerous health benefits of conducting regular physical activity among older adults. The built environment is believed to be a key factor that can hinder or facilitate daily physical activity, such as walking and exercising. However, most empirical studies focusing on environment-physical activity associations exhibited residential self-selection bias with cross-sectional research design, engendering doubts about the impact of built environment on physical activity. To reduce this bias, we assessed physical activity behaviors of 720 Hong Kong older adults (≥65 years) residing in 24 public housing estates. The Hong Kong public housing scheme currently provides affordable rental flats for 2.1 million people or approximate 30% of total population. The applicants were allocated to one of 179 housing estates largely by family size and flat availability. Built environment characteristics were measured following the ‘5Ds’ principle: (street network) design, (land-use) diversity, density, distance to transit, and destination accessibility. Multilevel mixed models were used to explore the associations between the built environment and the different domains of physical activity (transportation walking, recreational walking, and recreational moderate-to-vigorous physical activity (MVPA) while controlling for potential estate-level socioeconomic and individual confounders. We found that transportation walking was positively associated with the number of bus stops and the presence of Mass Transit Railway (MTR) stations. Recreational MVPA was positively related to the number of recreational facilities. However, land-use mix was negatively related to transportation walking, recreational walking, and recreational MVPA. The findings of this study support a threshold effect in the environment-physical activity associations. Furthermore, large-scale public housing schemes involving random or semi-random residence assignment in many cities may provide opportunities to explore built environments and physical activity behavior, with the potential to overcome residential self-selection bias.

## 1. Background

Hong Kong and many global cities are facing the challenges of aging populations, with one challenge being an increased demand for healthcare services and expenditure. Hong Kong is one of the fastest-aging societies. By 2031, one in every four Hong Kong residents is expected to be 65 years of age or over [1].

There is compelling evidence that conducting regular physical activity improves the physical and mental health of older adults in various aspects. Regular walking or exercise reduces the risk of obesity, type II diabetes, cardiovascular diseases, depression, falls, and anxiety [2,3]. Public health officials and researchers from various fields are exploring the role of the built environment in promoting physical activity. Research has shown that older adults are more active in neighborhoods with certain built environment attributes. Those attributes include the proximity to various destinations, street connectivity, availability of paths for walking, availability of public transportation facilities, environmental aesthetics, and safety [4,5,6,7,8,9]. For example, access to parks or recreational facilities in neighborhoods is positively related to higher rates of transport walking for older adults living in retirement villages [10]. Walking-friendly infrastructure—such as provision of well-maintained sidewalks, street lights, benches along streets, and street trees—was also found to be positively related to total physical activity and walking among older adults [8]. In addition, personal safety from crime, especially when it is self-reported, was related to physical activity walking for older adults; while traffic safety was unrelated [8]. Hence, interventions to improve walking infrastructure, provide parks and recreational facilities, and improve personal safety may promote overall physical activity and walking behaviors for older adults [4,8].

Some studies further pinpointed built environment characteristics within a ‘3Ds’ framework, which was then expanded to a ‘5Ds’ framework [11,12]. These frameworks become effective international systems for classifying and estimating built environment characteristics in active living research. The original 3Ds are (land use) diversity, urban density, and (street network) design. The two additional Ds are distance to transit and destination accessibility. These attributes showed confirmed associations with physical activity for older adults, especially in Western countries [4,5,6]. For example, walkability, a composite score of the 3Ds, was positively related to transportation walking and MVPA for older adults in two areas of the USA [13,14]. Patterson and Chapman reported that urban designs with high levels of land-use mix could promote walking for older adults in Portland, Oregon, USA [15].

**Figure 1:**
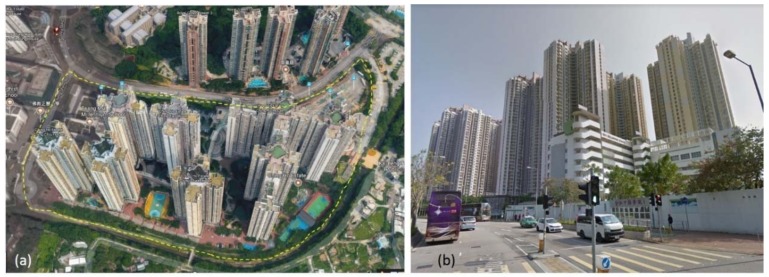
Aerial image (**a**) and street-level image (**b**) of one public housing estate in Hong Kong (Ching Ho Estate, built in 2006 with 7200 rental flats). Public housing estates generally consist of multiple 30-to 40-story residential towers and surrounding public spaces built for physical and leisure activities (Source: Google maps, 2016).

There are two caveats in the literature. First, most evidence came from Western countries, especially North America and Australia [4,7,8]. A recent review calls for studies from diverse regions to understand the complex patterns in built environment-physical activity associations [7]. Recent findings from Asia and South America showed nonsignificant or even negative results between the 3Ds variables and walking behavior [16,17,18,19,20]. In Zhongshan city, China, population density and land-use mix were negatively associated to the number of walking trips for 4308 older Chinese adults (aged 60 years or above) [20]. The urban density was reported to be negatively linked to recreational walking for women, in another dense city in China, Hangzhou [17]. Similar negative results were reported in South America. Residential density was found to be negatively linked to the objectively measured MVPA for 677 residents In Cuernavaca, Mexico [19]. The contrasting findings from different countries support the built environment-physical activity associations may be moderated by countries, i.e. those associations differ across different countries given the heterogeneity in the built and social environments across countries [21]. Hong Kong, a densely populated metropolitan of China, is especially understudied. In fact, the urban areas of Hong Kong are much denser than those in North America and Australia (Figure 1). The car ownership is only 0.07 per person in Hong Kong, lower than most Western countries [1]. As we know, only one study focusing older adults’ physical activity and objective built environmental features [22]. That study reported that recreational physical activity was positively related to availability of recreational facilities and safety in neighborhoods for 484 older adults [22]. Yet, this study did not consider walking, an important domain of physical activity.

Second, most empirical studies carried out to clarify this relationship have used a cross-sectional research design, which makes the observed built environment-physical activity associations uncertain [23,24,25]. Residential self-selection bias may severely undermine those reported associations [24]. For instance, an older adult enjoying walking and having positive belief on physical activity may decide to move to a neighborhood with walking-supportive characteristics. Hence, the observed environment–behavior relationships may be accounted for by personal lifestyle rather than by causal relationships [25].

Several research design options were implemented to mitigate this bias, albeit without focus on older adults. Some researchers suggested measuring personal preferences and attitudes and then control those personal factors as confounders during data analysis. One study found that built environment had no significant influence on walking when including personal attitudes and preferences in statistical models [26]. On the other hand, some other researchers demonstrated that the built environment still exerted a moderate effect on walking even after considering personal attitudes and preferences [27]. Yet, the estimating of personal attitudes and preference by self-reporting method, however, may be unreliable [28,29].

Other researchers proposed longitudinal research design by suggesting that personal attitudes and preferences are same before and after residential relocations or built environment interventions [27,29]. Therefore, the change in behaviors are largely due to the change in the built environment characteristics [28,29]. Krizek [30] assessed the change in travel behaviors before and after households’ relocation and reported that households moving to neighborhood with higher density and land use mix reduced vehicle use [30].

The large-scale Hong Kong public housing system offers an alternative research opportunity to address the self-selection bias. Hong Kong government currently provides about 0.8 million affordable rental flats for 2.1 million low-income people as a long-established safety net [31]. The applicants were allocated to one of 179 housing estates throughout the whole Hong Kong territory largely by family size and flat availability. Hence, we can largely rule out personal attitudes and preferences, and reduce residential self-selection bias. Furthermore, approximately 56% of older adults in Hong Kong reside in public housing estates [31]; improving existing or designing new public housing estates to promote physical activity may have a positive impact for the majority of older adults in Hong Kong.

In the present study, we compared physical activity behaviors of 720 Hong Kong older adults (≥65 years) residing in 24 public housing estates to reduce the self-selection bias. Furthermore, objective built environment characteristics for those 24 selected housing estates were assessed with Geographic Information System (GIS) obtained from the Hong Kong government.

## 2. Methods

Hong Kong is a coastal city in the developed area of China; it features high-density urban development and affordable public housing system. The gross population density of Hong Kong in 2016 was 6603 people per km^2^; private automobile ownership is only 0.07 per resident [1].

Hong Kong achieved international renown for the large-scale public housing system, which provide inexpensive accommodation for low-income families. As of 2016, there were a total 179 public housing estates and a total stock of 0.8 million flats. One public house estate, on average, has 3975 housing flats and 60,000 m^2^ site area, accommodating 10,832 residents [31]. Public housing estates generally consist of multiple 30- to 40-story residential towers and surrounding public spaces built for physical and leisure activities, often including basketball courts, playgrounds, and elderly fitness centers [32,33] (Figure 1). The housing estate is the also smallest census unit in Hong Kong. The 179 public housing estates constituted a base for selecting 24 study areas in this study.

### 2.1. Sample Areas

There were two criteria to select 24 public housing estates: estate-level household income (low or high) and walkability (low or high), because both criteria have significant relationships with physical activity in previous studies [34,35,36,37,38].

The walkability of each public housing estate was measured by a walkability index, comprised of three measures: (1) population density, (2) street connectivity, and (3) land-use mix [37,39]. The index was calculated for a 1 km street-network buffer around the center of a public housing estate. The 1 km radius was determined to be the average walking trip distance for older Chinese adults, given an average duration of 14 min per walking trip and a 70 m/min walking speed [20,40]. This radius was also used in other international studies [21,41]. Among three measures, the population density data were available from Census & Statistics Department of Hong Kong government. The density was measured by the residential population per km^2^ (people/km^2^). Land-use mix was measured by the land-use entropy score, indicating the level of land-use diversity [37,42]. Four land-use types were included in entropy score: residential, retail, office, and recreational. Street connectivity was calculated as the number of three-way or four-way street intersections per km^2^ in the buffer (count of intersections/km^2^). The street intersection data were also available from Planning Department of Hong Kong. Denser street intersections means high street connectivity by providing more direct and shorter routes to reach destinations, which may promote walking. The walkability index for each estate was measured with the following formula, where *Z* indicates *z*-score [39]

 Walkability index=Z (residential density)+Z (landuse mix)+2×Z (intersection density) 

Public housing estates were divided into ten groups with equal size by the walkability index. We labeled housing estates in the second to fourth deciles as low walkability and those in the seventh to ninth deciles as high walkability. The selection procedure is in line with the previous study [36].

The median household income of each housing estate was used to measure estate-level household income, and it was obtained from the Census & Statistics Department. All public housing estates were divided into two equal-sized parts: the lower part represented low-income housing estates and the higher half represented high-income housing estates.

All public housing estates in Hong Kong were classified by walkability and estate-level income. We selected a total of 24 public housing estates classified into four groups with six estates in each group: high/high (income/walkability respectively), high/low, low/high, and low/low. Additional efforts were made to assure equal numbers of housing estates within and beyond a 1-km walking distance from the nearest Mass Transit Railway (MTR) station because MTR is the most reliable and most used transport mode in Hong Kong.

### 2.2. Participants

We interviewed a total of 720 older adults in 2016, with 30 older adults from each of the 24 housing estates. The sample size was based on previous research [38,43]. All participants were Chinese adults aged 65 or above who could walk independently, and they should be reside in the current public housing estates longer than 12 months. Ethical approval was obtained prior to this study (no. H000691, City University of Hong Kong).

### 2.3. Physical Activity Data

A modified short version of the International Physical Activity Questionnaire (IPAQ) was used to assess three domains of physical activity: transportation walking, recreational walking, and recreational moderate-to-vigorous physical activity (MVPA). The IPAQ is an international questionnaire demonstrating robust test–retest reliability for many countries [44]. The IPAQ was conducted through face-to-face interviews. Total minutes of transportation walking, recreational walking, and recreational MVPA during the last seven days were recalled with IPAQ. Because total minutes of transportation walking, recreational walking, and recreational MVPA were not normally distributed, they were transformed into two-level categorical variables (≥150 min/week or <150 min/week). The cut-off of 150 min/week was based on previous studies’ recommendations [45,46].

### 2.4. Objective Measurements of the Built Environment

We also measured the other two ‘D’s: distance to transit and destination accessibility [46]. The destination accessibility was assessed by the number of retail shops—including shopping malls, supermarkets, markets, and convenience stores—and the number of recreational facilities—including parks, recreational and sports facilities within the buffer. Distance to transit was measured by the number of bus stops and the presence of MTR stations within the 1-km buffer.

### 2.5. Covariates

Some personal or household characteristics including gender, age, and monthly household income, were collected in the interview and used as covariates. The estate-level covariate included housing estate median household income. These covariates were included because the demographics and SES factors may also affect physical activity [36,38].

### 2.6. Data Analysis

Descriptive statistics of built environment and participants’ characteristics were analyzed using SPSS 21.0 (IBM Corp., Armonk, NY, USA). Means (M) and standard deviations (SD) were reported for continuous variables, whereas percentages and counts were reported for categorical variables.

In this study, three domains of physical activity were measured as binary outcomes: transportation walking, recreational walking, and recreational MVPA. Three multilevel logistic models were conducted in R with lme4 package to explore the relationships of built environment characteristics with the odds of conducting regular transportation walking (≥150 min/week), recreational walking, and recreational MVPA respectively, after controlling for individual and estate-level covariates. Two-level modeling (participant–housing estate) was applied to consider the clustering of participants’ physical activity behaviors in housing estates. The intra-class correlation (ICC), the ratio of between-cluster variance to the total variance, was used to show the proportion of total variance in the outcomes that is accounted for by the clustering, and to show to goodness of fit of multilevel analysis.

To interpret the odds ratios with ease, all continuous built environment variables were converted to two-level categorical variables (low vs. high) based on their median value, with the low level as the reference. These variables included street intersection density, land-use mix, population density, number of bus stops, number of retail shops, and number of recreational facilities. Presence of MTR station was coded as a two-level categorical variable (no vs. yes). Individual and estate-level covariates controlled in the models included gender, age, participant’s household income, and median household income of a housing estate. The participants’ age was transformed into a two-level categorical variable (65 to 74 years, and ≥75 years). Their monthly household income was also transformed into a categorical variable (<HK$12,000, and ≥HK$12,000).

## 3. Results

### 3.1. Built Environment and Participants’ Characteristics

The 24 selected public housing estates featured high levels of 5D variables, e.g., high population densities (M = 74,911 people/km^2^, SD = 19,273), highly mixed land-use (M = 0.37, SD = 0.31), and great availability of bus stops (M = 45, SD = 31), and recreational facilities (M = 31, SD = 22) (Table 1).

Overall, 35.4% of older adults achieved at least 150 min of transportation walking, 58.9% achieved at least 150 min of recreational walking, and 38.5% achieved at least 150 min of recreational MVPA in one week (Table 2). Recreational walking was the most popular domain of physical activity for older adults in Hong Kong. There were more women than men in our study (52.1% versus 47.9%), matching the gender distribution of the Hong Kong population (53.2% versus 46.7%) [1]. Proportionally, women were slightly more active than men. The median household income of our participants was lower compared with population-level median income, given that we only sampled older adults living in public housing estates.

### 3.2. Association between Built Environment Characteristics and Physical Activity

Table 3 shows the results of a generalized mixed model with the occurrence of regular transportation walking (≥150 min), regular recreational walking (≥150 min), and regular recreational MVPA (≥150 min) as the outcomes. The intra-class correlations (ICCs) of the intercept-only models for the three outcomes (transportation walking, recreational walking, and recreational MVPA) were 0.307, 0.239, and 0.209 respectively. The ICCs for the full models using the three outcomes were reduced to 0.245, 0.153, and 0.117 respectively.

Transportation walking was negatively related to land-use mix. Older adults living in neighborhoods with higher land-use diversity had lower odds of achieving regular transportation walking than those living in neighborhoods with lower land-use mix (OR = 0.87, CI = 0.78–0.96, *p* < 0.01). Transportation walking was positively associated with the fifth D, distance to transit—measured by the number of bus stops and the presence of an MTR station. Older adults living in neighborhoods with an MTR station had higher odds of achieving regular transportation walking than those living in neighborhoods without an MTR station (OR = 1.20, CI = 1.04–1.38, *p* < 0.01). Similarly, older adults living in neighborhoods with more bus stops had higher odds than those living in neighborhoods with fewer bus stops. Transportation walking was not associated with the remaining built environment variables.

Recreational walking was negatively related to land-use mix and the number of bus stops. Older adults living in neighborhoods with higher land-use mix had lower odds of achieving regular recreational walking compared with those living in neighborhoods with lower land-use mix (OR = 0.85, CI = 0.73–0.95, *p* < 0.01). Older adults living in neighborhoods with more bus stops also had lower odds of achieving regular recreational walking compared with those living in neighborhoods with fewer bus stops (OR = 0.98, CI = 0.96–0.99, *p* = 0.02). Recreational walking was not associated with the remaining built environment variables.

Recreational MVPA was negatively related to land-use mix and the number of bus stops. Older adults living in neighborhoods with higher land-use mix had lower odds of achieving regular recreational MVPA compared with those living in neighborhoods with lower land-use mix (OR = 0.78, CI = 0.64–0.91, *p* < 0.01). Older adults living in neighborhoods with more bus stops also had lower odds of achieving regular recreational MVPA compared with those living in neighborhoods with fewer bus stops (OR = 0.96, CI = 0.95–0.97, *p* < 0.01), whereas recreational MVPA was positively associated with the number of recreational facilities. Older adults living in neighborhoods with more recreational facilities had higher odds of achieving regular recreational MVPA than those living in neighborhoods with fewer recreational facilities (OR = 1.11, CI = 1.04–1.18, *p* < 0.01). Recreational MVPA was not associated with the remaining built environment variables.

## 4. Discussion

In this study, we examined the association between built environment characteristics and three domains of physical activity for 720 Hong Kong older adults living in 24 public housing estates, while controlling for potential covariates—age, gender, individual household income, and estate-level SES. There were two main findings.

First, we found some positive associations between physical activity and the additional 2Ds of built environment characteristics: destination accessibility (number of recreational facilities) and distance to transit (number of bus stops and the presence of an MTR). Older adults in neighborhoods with MTR presence and more bus stops had a higher chance of achieving regular transportation walking. Older adults in neighborhoods with more recreational facilities had a higher chance to achieve regular MVPA.

These results concur with those of several empirical studies [15,22,43,47,48,49]. Residential neighborhoods with better access to services and shops within 1 mile from home were associated with increased walking among 133 older women over 70 years of age in Portland, Oregon, USA [15]. Older adults (*n* = 2918) with access to general services and social infrastructure were more likely to do some walking in the Perth metropolitan region in Australia [49]. The number of bus stops and easy access to commercial establishments were related to more transportation walking trips for older adults over 60 years of age in the Zhongshan Metropolitan Area in China [20]. These findings, together with those of this study, suggest that easy access to public transit (both bus and MTR) may increase transportation walking while easy access to recreational facilities may increase physical activity for older adults [5,6].

Second, we also found largely negative and insignificant associations between physical activity and the original 3Ds—diversity, design, and density. Land-use mix was negatively associated with the odds of engaging in regular transportation walking, recreational walking, or recreational MVPA. Furthermore, street connectivity and population density are not related to any domains of physical activity. Those negative and insignificant associations founded in this study markedly differ from those observed in Western countries.

There may be three reasons for the contrasting associations found in Hong Kong and its Western counterparts. First, Hong Kong neighborhoods typically are more densely and diversely developed compared with urban areas in the Australia or USA. Public housing estates, for example, are typically built in the form of multiple 30- to 40-story towers. The average population density was 74,911 people/km^2^ in our 24 study areas, which is approximately 50 times higher than those in Atlanta region, USA [13] and 250 times higher than that in Perth, Australia [49]. Therefore, the low-density neighborhoods in Hong Kong are in fact denser than the high-density neighborhoods in the USA or Australia. The same applies to land-use mix. The different ranges of urban density and land-use mix may account for the contrasting findings. Similar negative associations were reported for adults in Hong Kong [50,51].

Second, older adults in Hong Kong rely heavily on walking and public transportation systems to access their daily destinations, such as supermarkets, restaurants, or recreational facilities. The overall vehicle ownership per person is only 0.07 as of 2016, which is much lower than 0.79 in the US or 0.74 in Australia. In Hong Kong, the relatively low urban density and land-use mix may make daily destinations further away from home and thus promote longer walking trips because few older adults drive. In sum, the unique local built environment contexts in Hong Kong—the extremely high diversity, highly dense development, and heavily reliance on public transportation systems—may contribute to the insignificant or negative correlations between the original 3Ds and older adults’ physical activity.

Third, the associations of 3Ds and physical activity may be moderated by other social or built environment factors, such as personal safety [4,7,22,52] or street greenery [53,54]. For example, previous studies demonstrated strong evidence between personal safety and physical activity or walking. Older adults who perceived their neighborhoods to be safer tended to walk more often than others [8]. It is plausible that older adults in Hong Kong consider areas with higher density and land-use mix to be less safe and hence are more concerned with personal safety, which hinders their physical activity. Yet, the safety data in small administrative units were unavailable from Hong Kong government. Future studies with safety data, either objective measured or self-reported, should be included.

These mixed results suggest a threshold effect in the associations between the 3Ds and physical activity [21]. Our results showed that land-use mix was negatively related to walking and recreational MVPA. Those findings carry crucial urban planning implications for Hong Kong government, who should examine built environment–physical activity associations in local contexts, because learning lessons from other counties may be ineffective. Researchers in Western countries often advocate for high diversity and high-density urban development to encourage residents to be physically active. This proposition may be unproductive for Hong Kong, a city already achieving high density and diversity in urban development. Urban planners and government agencies should redirect their attention on macro-scale planning parameters (e.g., density, street connectivity, and land-use) to micro-scale urban design features (e.g., easy access to pedestrian destinations and public transit). Our suggestion is that by paying more attention to the latter, we may improve current built environment conditions to provide greater health benefits for older adults in Hong Kong.

This study had both strengths and limitations. First, in terms of its strengths, it used large-scale public housing system to reduce self-selection bias. The public housing scheme in Hong Kong allocates applicants to different neighborhoods mainly by applicant’s family size and housing availability. In principle, public housing flats are allocated to applicants according the order of their applications. The allocation was done with random computer batching for equitable distribution of public housing resources. This allocating procedure largely separates the effects of inter-personal preferences from those of built environment on physical activity. Observing residents’ physical activity in large-scale public housing schemes provided an alternative research opportunity to reduce residential self-selection bias. Another strength was that we objectively assessed the built environment with GIS data from the Hong Kong government, which supports replication of this study.

This study also had some limitations. Though self-selection bias is not a major issue in public housing allocation system, other types of bias may exist. For example, due to the completion of a new housing estate with thousands of flats, many households will move into it at the same time. Therefore, research with longitudinal design is still encouraged. In addition, physical activity was self-reported and thus prone to recall error. Future studies that objectively assess physical activity behaviors with accelerometers and/or global positioning system (GPS) devices are needed to help us understand the effects of built environment characteristics on physical activity with a higher degree of certainty. The demographic characteristics—e.g., gender and age—were treated as confounding factors for physical activity. Further studies may examine how the built environment–physical activity association is moderated by demographic characteristics. By doing so, we may implement targeted environmental interventions for specific demographic groups. The study sampling in the present study was limited to low-income older adults living in public housing flats. Thus, the generalizability of these findings is limited by the sampling population. In other words, the findings may not pertain to more affluent older adults living in private housing estates. Moreover, in Hong Kong and many Chinese cities, the well-established public transportation system comprises a variety of mode choices: bus, subway, minibus, tram, and light rail train. Future studies should also pay attention to the choice among different active travel modes rather than the choice between active and motorized travel mode.

## 5. Conclusions

Promoting physical activity to maintain health among older adults is a public health priority in Hong Kong and many cities worldwide. To reduce the impact of residential self-selection bias, we compared the physical activity behaviors of 720 older adults living in 24 public housing estates in Hong Kong, China. We confirmed that additional 2Ds of built environment characteristics—easy access to public transit and pedestrian destinations—were associated with increased walking and MVPA for older adults. The original ‘3Ds’ variables—density, land-use mix, design—however, were largely negative or not associated with any specific domains of physical activity. The mixed results from this study and others show a threshold effect in the built environment-physical activity associations.

## Figures and Tables

**Table 1:** Built environment characteristics of 24 public housing estates in Hong Kong, 2016.

5D Framework	Built Environment Variables	Mean (SD)
Density	Population density-all (persons/km^2^)	74,911 (19,273)
	Low (<76,871)	59,500 (10,657)
	High (≥76,871)	90,322 (11,139)
Diversity	Land-use mix (entropy score)	0.50 (0.31)
	Low (<0.37)	0.26 (0.05)
	High (≥0.37)	0.75 (0.26)
Design	Street intersection density-all (#/km^2^)	84.71 (57.67)
	Low (<102)	79.81 (21.54)
	High (≥102)	127.16 (27.30)
Distance to transit	No. of bus stops-all	45.13 (31.04)
	Low (<34)	19.67 (8.35)
	High (≥34)	70.48 (21.97)
	Presence of MTR station	No. of estates (%)
	No (0)	12 (50%)
	Yes (1)	12 (50%)
Destination accessibility	No. of retail shops-all	27.08 (17.11)
	Low (<22)	13.38 (5.32)
	High (≥22)	40.58 (12.99)
	No. of recreational facilities-all	31.54 (22.24)
	Low (<29)	14.17 (7.67)
	High (≥29)	48.92 (16.92)

**Table 2:** Characteristics of the 720 study participants residing in 24 public housing estates in Hong Kong, 2016.

Variables	No. of Participants (%)	No. of Participants Achieving ≥150 min Transportation Walking (%)	No. of Participants Achieving ≥150 min Recreational Walking (%)	No. of Participants Achieving ≥150 min MVPA Walking (%)
All	720 (100%)	255 (35.4%)	424 (58.9%)	277 (38.5%)
Gender				
Male	345 (47.9%)	115 (33.3%)	201 (58.3%)	130 (37.7%)
Female	375 (52.1%)	140 (37.3%)	223 (59.5%)	147 (39.2%)
Household income				
<HK$12,000	439 (61.0%)	156 (35.5%)	257 (58.5%)	155 (35.3%)
≥HK$12,000	281 (39.0%)	99 (35.2%)	167 (59.4%)	122 (43.4%)
Age				
65–74	512 (71.1%)	185 (36.1%)	297 (58.0%)	204 (39.8%)
≥75	208 (28.9%)	70 (33.7%)	127 (61.1%)	73 (35.1%)

**Table 3:** Multilevel logistic regression of built environment factors and the odds of achieving regular transportation walking (≥150 min), recreational walking (≥150 min), and recreational MVPA (≥150 min) for 720 older adults in Hong Kong, while controlling for other covariates.

Built Environment Factors	Transportation Walking		Recreational Walking		Recreational MVPA	
	OR (95% CI)	*p*	OR (95% CI)	*p*	OR (95% CI)	*p*
Intersection density						
Low (<102)	Ref.		Ref.		Ref.	
High (≥102)	1.23 (0.82–1.85)	0.31	1.07 (0.73–1.59)	0.71	0.97 (0.65–1.45)	0.89
Population density						
Low (<76,871)	Ref.		Ref.		Ref.	
High (≥76,871)	1.45 (0.90–2.11)	0.06	1.00 (0.70–1.42)	0.99	0.82 (0.56–1.18)	0.28
Land-use mix						
Low (<0.37)	Ref.		Ref.		Ref.	
High (≥0.37)	0.87 (0.78–0.96)	<0.01 **	0.85 (0.73–0.95)	<0.01 **	0.78 (0.64–0.91)	<0.01 **
No. of bus stops						
Low (<34)	Ref.		Ref.		Ref.	
High (≥34)	1.03 (1.01–1.05)	<0.01 **	0.98 (0.96–0.99)	0.02 *	0.96 (0.95–0.97)	<0.01 **
Presence of MTR station						
No (0)	Ref.		Ref.		Ref.	
Yes (1)	1.20 (1.04–1.38)	<0.01 **	1.03 (0.89–1.38)	0.13	0.91 (0.79–1.05)	0.22
No. of retail shops						
Low (<22)	Ref.		Ref.		Ref.	
High (≥22)	0.98 (0.86–1.09)	0.19	0.89 (0.63–1.25)	0.50	0.96 (0.92–1.02)	0.06
No. of recreational facilities						
Low (<29)	Ref.		Ref.		Ref.	
High (≥29)	1.01 (0.88–1.12)	0.21	1.01 (0.99–1.02)	0.21	1.11 (1.04–1.18)	<0.01 **

Note 1: ** *p* < 0.01, * *p* < 0.05. OR = Odds ratio, CI = Confidential interval. Note 2: Individual and estate-level covariates controlled in the models included: gender, age, participant’s household income and estate-level median household income. All built environment variables were entered the models simultaneously.
